# Effect of Brazilian Propolis on Exacerbation of Respiratory Syncytial Virus Infection in Mice Exposed to Tetrabromobisphenol A, a Brominated Flame Retardant

**DOI:** 10.1155/2013/698206

**Published:** 2013-10-22

**Authors:** Tomomi Takeshita, Wataru Watanabe, Satomi Toyama, Yuya Hayashi, Shiori Honda, Shuichi Sakamoto, Sayuri Matsuoka, Hiroki Yoshida, Shiro Takeda, Muneaki Hidaka, Shigetoshi Tsutsumi, Ken Yasukawa, Yong Kun Park, Masahiko Kurokawa

**Affiliations:** ^1^Department of Biochemistry, Graduate School of Clinical Pharmacy, Kyushu University of Health and Welfare, 1714-1 Yoshino, Miyazaki, Nobeoka 882-8508, Japan; ^2^Department of Microbiology, Graduate School of Clinical Pharmacy, Kyushu University of Health and Welfare, 1714-1 Yoshino, Miyazaki, Nobeoka 882-8508, Japan; ^3^Research Division, Minami Nihon Rakuno Kyodo Co. Ltd., Miyazaki, Miyakonojo 885-0003, Japan; ^4^Clinical Pharmacy, Graduate School of Clinical Pharmacy, Kyushu University of Health and Welfare, 1714-1 Yoshino, Miyazaki, Nobeoka 882-8508, Japan; ^5^Amazonfood Ltd., 3-1-8 Misaki, Chiyoda, Tokyo 101-0061, Japan; ^6^School of Pharmacy, Nihon University, 7-7-1 Narashinodai, Chiba, Funabashi-shi 274-8555, Japan; ^7^Department of Food Science, College of Food Engineering, State University of Campinas, P.O. Box 6177, 13083-970 Campinas, SP, Brazil

## Abstract

Tetrabromobisphenol A (TBBPA), a brominated flame retardant, has been found to exacerbate pneumonia in respiratory syncytial virus- (RSV-) infected mice. We examined the effect of Brazilian propolis (AF-08) on the exacerbation of RSV infection by TBBPA exposure in mice. Mice were fed a powdered diet mixed with 1% TBBPA alone, 0.02% AF-08 alone, or 1% TBBPA and 0.02% AF-08 for four weeks and then intranasally infected with RSV. TBBPA exposure increased the pulmonary virus titer and level of IFN-**γ**, a representative marker of pneumonia due to RSV infection, in the lungs of infected mice without toxicity. AF-08 was significantly effective in reducing the virus titers and IFN-**γ** level increased by TBBPA exposure. Also, AF-08 significantly reduced proinflammatory cytokine (TNF-**α** and IL-6) levels in the lungs of RSV-infected mice with TBBPA exposure, but Th2 cytokine (IL-4 and IL-10) levels were not evidently increased. Neither TBBPA exposure nor AF-08 treatment affected the anti-RSV antibody production in RSV-infected mice. In flow cytometry analysis, AF-08 seemed to be effective in reducing the ratio of pulmonary CD8a^+^ cells in RSV-infected mice with TBBPA exposure. TBBPA and AF-08 did not exhibit anti-RSV activity *in vitro*. Thus, AF-08 probably ameliorated pneumonia exacerbated by TBBPA exposure in RSV-infected mice by limiting excess cellular immune responses.

## 1. Introduction

Propolis has been used worldwide as a folk medicine since ca. 300 BC and as a dietary supplement to maintain or improve human health [1–3]; it is currently used as an alternative medicine in the management of various ailments [4]. Propolis is a resinous hive product and consists of a mixture of plant exudates collected by honeybees and beeswax. The ethanol extract of Brazilian propolis (AF-08) has been shown to exhibit anti-influenza virus activity *in vitro* and *in vivo* and ameliorate influenza symptoms in mice [5]. We also showed that AF-08 has an immunomodulatory activity against intra-dermal herpes simplex virus type-1 (HSV-1) infection in mice and ameliorates herpes symptoms in mice, although it has no anti-HSV-1 activity *in vitro* [6]. The immunomodulatory activity associated with interferon- (IFN-) *γ* production inducing Th1 immunity in mice was suggested to contribute to the elucidation of various pharmacological actions of propolis in health and disease [6].

Human respiratory syncytial virus (RSV), a member of the *Paramyxoviridae* family, is the most prevalent infectious agent of acute lower respiratory illness in infants and young children [7]. Infection and reinfection with RSV are frequent during the first few years of life, and most children are infected by 24 months of age [8]. Clinically severe RSV infection is seen primarily in young children with naive immune systems and/or genetic predisposition [9], patients with suppressed T-cell immunity [7], and the elderly [10]. It is suggested that the severity of RSV infection is associated with immunological defect.

We have recently established a novel assay system for the evaluation of the developmental immunotoxicity of brominated flame retardants (BFRs) using a mouse model of RSV infection [11]. Using this model, perinatal exposure to BFRs has been shown to elevate the levels of IFN-*γ* in the bronchoalveolar lavage fluid (BALF) of RSV-infected offspring mice with an increase in pulmonary viral titers and exacerbate pneumonia [12, 13], indicating that BFRs are a risk factor for RSV infection across human generations. BFRs are ubiquitously used as industrial materials worldwide. They are used as additive or reactive components in a variety of polymers, such as polystyrene foams, high-impact polystyrene, and epoxy resins [14]. Notably, tetrabromobisphenol A (TBBPA) accounted for approximately 76% of the BFRs consumed in Asia in 2001, and the amount used was approximately 90,000 tons [14]. BFRs are easily released into an environment due to deterioration or abrasion of the materials and exist ubiquitously in the environment [15–17]. Some of them have been shown to cause endocrine disruption and reproductive damage and to be immunotoxic and neurotoxic [18–23]. They are suspected of being toxic to children [24]. Recently, we demonstrated changes in cytokine production and immune cell populations not only in offspring mice born to mice perinatally exposed to BFRs but also in normal mice exposed to BFRs, and the irregularities due to BFRs exposure have been suggested to exacerbate pneumonia in RSV-infected mice [25].

In this study, the effect of Brazilian propolis (AF-08) on the exacerbation of RSV infection in mice exposed to TBBPA was examined. We found that AF-08 alleviated the symptoms of pneumonia exacerbated by TBBPA exposure. The mode of alleviation was evaluated virologically and immunologically, and we characterized the potential and pharmacological activity of Brazilian propolis AF-08. 

## 2. Materials and Methods

### 2.1. Cell and Virus

Human epidermoid carcinoma (HEp-2) cells (American Type Culture Collection, CCL-23, Rockville, MD) were purchased from Dainippon Pharmaceutical (Osaka, Japan) and grown and maintained in Eagle's minimum essential medium supplemented with 10% and 2%, respectively, heat-inactivated fetal calf serum. The A2 strain of RSV was obtained from American Type Culture Collection (Rockville, MD) and grown in HEp-2 cell cultures. Viral titers of HEp-2 cell cultures were measured by the plaque method and expressed as plaque-forming units per milliliter (PFU/mL) [11].

### 2.2. TBBPA and Propolis

TBBPA (Mw: 543.87; purity: >93%) was purchased from Tokyo Kasei (Tokyo, Japan) and mixed into a powdered diet, which was soy-free to avoid the estrogen-like effect of soybeans, based on the formulation of the NIH-07 open-formula rodent diet [26] and produced by Oriental Yeast Co. (Chiba, Japan).

Brazilian propolis AF0307 was immersed in 95% ethanol (1 : 1, w/w) for 3 months with occasional mixing at room temperature in the dark to avoid UV/light exposure, and the ethanol extracts were dried in a vacuum oven at 37°C to 38°C for 40 to 60 min as described previously [6]. The voucher specimen of AF0307 was deposited at Amazonfood Co., Ltd., Tokyo, Japan. The ethanol extract (AF-08) was supplied by Amazonfood Co., Ltd., Tokyo, Japan. AF-08 was dissolved in dimethyl sulfoxide (DMSO) at 100 mg/mL as a stock solution and diluted with culture medium to make various final concentrations for *in vitro* assays. The concentration of DMSO in each medium was less than 0.2%. For *in vivo* assays, a powdered soy-free diet that had already been mixed with 1% TBBPA (w/w) was further mixed with AF-08 at 0.02% (w/w).

### 2.3. Mice

Female BALB/c mice (4 weeks old) were purchased from Kyudo Animal Laboratory (Kumamoto, Japan). The mice were housed 5 to 7 per cage in specific pathogen-free conditions, with a conventional solid diet CRF-1 (Oriental Yeast Co.) and water *ad libitum* and under a 12 h light/12 h dark diurnal cycle (light at 7.00 a.m.). The temperature in the room was kept at 24 ± 2°C. The mice were acclimated for 7 days before starting experimental procedures. Animal studies followed the animal experimentation guidelines of Kyusyu University of Health and Welfare and were carried out in an approved biosafety level facility.

### 2.4. Murine RSV Infection

Five-week-old mice were fed a soy-free diet mixed with 1% (w/w) TBBPA alone, 0.02% (w/w) AF-08 alone, or 1% TBBPA and 0.02% AF-08 for 4 weeks. As a control, the mice were fed a soy-free diet without 1% TBBPA and 0.02% AF-08. After treatment, these mice were fed CRF-1 and used for the subsequent RSV infection experiments. Throughout the experiments, both chow and water were provided for *ad libitum* consumption. The RSV infection was performed as reported previously [25]. Briefly, 9-week-old female mice were infected intranasally with 1 × 10^6^ PFU per 0.1 mL of RSV under anesthesia. Mock-infected mice were also inoculated intranasally with 0.1 mL of phosphate-buffered saline (PBS) under anesthesia. On day 5 after infection, sera were obtained from the mice under anesthesia, and BALF was also obtained from the mice by instilling 1.0 mL of cold PBS into the lungs and aspirating it from the trachea using a tracheal cannula on days 1 and 5 after infection [27]. Ice-cold BALF was centrifuged at 100 ×g at 4°C for 10 min. After centrifugation, the supernatant was stored at −80°C until use. The cell pellets were suspended in PBS on ice and used as bronchoalveolar lavage (BAL) cells for flow cytometric analysis. For virus titration, the lungs were removed on day 5, immediately frozen in liquid N_2_, and stored at −80°C. Frozen lung tissue was homogenized with cold quartz sand in a homogenizer, and viral titers in the supernatants of the homogenates were measured by a plaque assay [25].

### 2.5. Plaque Reduction Assay and Cytotoxicity Assay

The anti-RSV activities of TBBPA and AF-08 were examined by a plaque reduction assay using HEp-2 cells [11]. Briefly, HEp-2 cells grown in 24-well plates were infected with 100 PFU/0.2 mL of RSV at 37°C for 1 h. The cells were overlaid with 1 mL of maintenance medium containing 0.8% methylcellulose and various concentrations of TBBPA, AF-08, or ribavirin and maintained in a humidified atmosphere containing 5% CO_2_ for 4-5 days. The infected cells were fixed and stained, and the plaques were counted [11]. TBBPA and AF-08 were dissolved in DMSO and diluted with culture medium to make the various final concentrations. The concentration of DMSO in each medium was less than 0.5%. Ribavirin was dissolved in distilled water and used as a control. In the plaque reduction assay, the visible cytotoxicity of TBBPA and AF-08 was evaluated by the extent of absence of uninfected cells on the surface of stained dishes, and strong cytotoxicity (++) was scored as the absence of more than 50% of uninfected cells as compared with untreated dishes (controls) as described previously [28].

 Cytotoxicities of TBBPA, AF-08, and ribavirin were assessed by MTT (3-[4,5-dimethylthiazol-2-yl]-2,5-diphenyltetrazolium bromide, Sigma, Tokyo, Japan) assay using HEp-2 cells in 96-well plates as described previously [29]. Briefly, HEp-2 cells grown in 96-well plates were overlaid with 50 *μ*L of maintenance medium containing various concentrations of the compounds and maintained in a humidified atmosphere containing 5% CO_2_ for 3 days. TBBPA and AF-08 were dissolved in DMSO and diluted with culture medium to make the various final concentrations. The concentration of DMSO in each medium was less than 0.5%. Ribavirin was dissolved in distilled water and used as a control. For the MTT assay, 10 *μ*L of MTT (7.5 mg/mL) solution in PBS was added to each well and incubated for 4 h at 37°C. The crystallized formazan in the plates was dissolved by the addition of 100 *μ*L of 20% (w/v) SDS/50% (v/v) N,N-dimethylformamide. Absorbance was measured at two wavelengths (540 and 690 nm) in a computer-controlled microplate reader (Bio-Rad, Tokyo, Japan) [29].

### 2.6. ELISA

*IFN-*γ**, tumor necrosis factor- (TNF-) *α*, interleukin- (IL-) 6, IL-4, and IL-10 levels in BALF were measured using a specific ELISA kit (Ready-Set-Go, eBioscience Inc., San Diego, CA) according to the manufacturer's instructions [25]. The product was tested and found to conform to all eBioscience Inc. Quality Control release specifications. The lower limits of detection sensitivity of the kits for IFN-*γ*, TNF-*α*, IL-6, IL-4, and IL-10 are 15, 8, 4, 4, and 30 pg/mL, respectively. The intra- and interassay coefficients of variation for these ELISA were less than 10%.

### 2.7. Flow Cytometric Analysis

Subpopulations of BAL cells were labeled with phycoerythrin- (PE-) labeled rat anti-CD8a (53.6) or rat-anti-CD11b (M1/70) antibodies (BD Bioscience Pharmingen, San Diego, CA) and analyzed by a FACS Calibur 3S flow cytometer (Becton Dickinson, Sunnyvale, CA). Briefly, BAL cells were collected from 4 to 5 mice in each group on days 1 and 5 after infection and pooled by group. The BAL cells (5 × 10^5^cells) were suspended in 50 *μ*L of PBS, mixed with 20 *μ*L of each PE-labeled antibody, and incubated at 4°C for 30 min. The cells were washed twice with PBS and at least 10,000 cells suspended in PBS were analyzed by FACS [25]. Finally, the data were analyzed with CellQuest software.

### 2.8. Determination of Anti-RSV Antibody Titer in Serum

 The anti-RSV antibody titer in sera was determined by the ELISA test. RSV antigen was prepared from RSV-infected HEp-2 cells, and the control antigen was simultaneously prepared from uninfected cells. The ELISA test was performed according to the methods described elsewhere [30]. Briefly, 96-well microtiter plates were coated with 100 *μ*L of RSV and control antigens diluted in PBS for 18 h at 4°C. The plates were washed and blocked, and then test sera diluted with PBS were incubated at 37°C for 1 h. Wells were washed extensively and incubated with peroxidase-conjugated rabbit anti-mouse IgG (DAKO A/S, Denmark) for 1 h at 37°C. After washes with PBS, the enzyme reaction was performed and then the absorbance of the reaction mixture was measured. Anti-RSV antibody titer was determined by comparing the absorbance of control and RSV antigens.

### 2.9. Statistical Analysis

Statistical significance of differences between virus titers, cytokine levels, and anti-RSV-antibody levels evaluated using Student's *t*-test. A *P* value of 0.05 or less was considered to be significant statistically.

## 3. Results

### 3.1. Effect of AF-08 on RSV Infection in Mice Exposed to TBBPA

**Figure 1:**
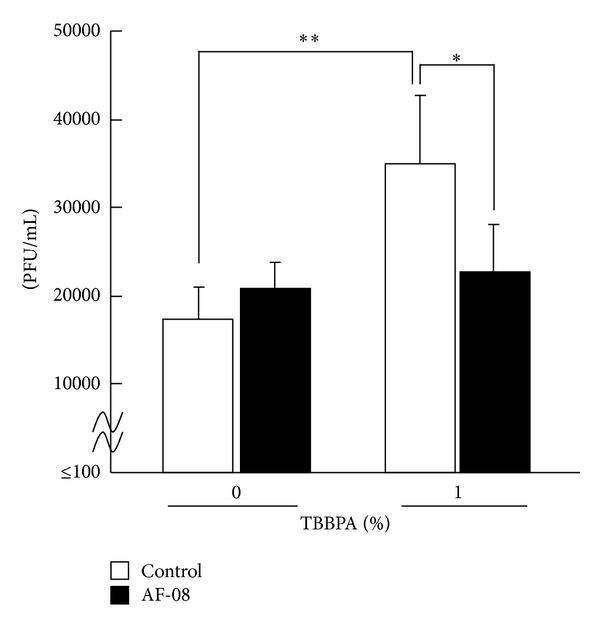
Effect of AF-08 on pulmonary viral titers in RSV-infected mice with TBBPA exposure. Seven mice in each group were fed a powdered diet mixed with 1% TBBPA alone, 0.02% AF-08 alone, or 1% TBBPA plus 0.02% AF-08 or without 1% TBBPA and 0.02% AF-08 (control) for 4 weeks and then infected with RSV at 10^6^ PFU/mouse. The lungs were removed on day 5 after infection, and virus titers in homogenized lungs were determined by a plaque assay. *P < 0.05 and **P < 0.001 versus control by Student's *t*-test. Bars indicate standard errors.

We previously showed that exposure to 1% TBBPA for 28 days significantly increased pulmonary virus titers in RSV-infected mice and exacerbated pneumonia histologically [25]. Using this murine RSV infection model, we examined the effect of AF-08 on the exacerbation of RSV infection due to TBBPA exposure. Five-week-old mice were fed a control or 1% TBBPA (w/w) diet for 28 days then intranasally infected with RSV. TBBPA-exposed mice showed no significant body weight loss or decrease in food consumption during the 28-day feeding period, when compared to control (data not shown). Then the mice with or without TBBPA exposure were intranasally infected with RSV. No significant weight loss was detected during the RSV infection phase (data not shown). On day 5 after infection, pulmonary viral titers in mice with TBBPA exposure were significantly higher than those in the control mice (*P* < 0.001 versus control by Student's *t*-test, Figure 1). We confirmed the absence of toxicity and increase in pulmonary virus titers in RSV-infected mice exposed to TBBPA as described previously [25].

 To examine the effect of AF-08 on the increase in pulmonary RSV titers by TBBPA exposure, we used 0.02% AF-08 mixed with a powdered diet with or without 1% TBBPA. In our previous studies, oral administration of AF-08 at 10 mg/kg, 3 times daily, was effective in reducing pulmonary influenza virus titers, resulting in the prolongation of survival times of influenza virus-infected mice [5] and in alleviating herpes simplex virus type-1 infection by enhancing delayed hypersensitivity to inactivate herpes simplex virus antigen [6]. These results indicate that 30 mg/kg/day may be a biologically active dose of AF-08 in mice. In addition, as the daily consumption of powered diet by a mouse is approximately 3 g, a powdered diet containing 0.02% AF-08 was deduced from 0.6 mg/mouse of 20 g/day corresponding to the dose of AF-08 (30 mg/kg/day) and a daily dose of approximately 1500 mg/kg/day TBBPA was estimated. In the AF-08-treated and AF-08-treated plus TBBPA-exposed mice, there was no significant body weight loss or decrease in food consumption compared with the control (data not shown). As shown in Figure 1, 0.02% AF-08 did not affect pulmonary virus titers in RSV-infected mice without TBBPA exposure but significantly reduced the virus titers in RSV-infected mice with TBBPA exposure (*P* < 0.05 versus TBBPA alone by Student's *t*-test). The reduced titer was similar to those in RSV-infected mice without TBBPA exposure. Thus, AF-08 was effective in reducing RSV titers increased by TBBPA exposure to the normal levels without TBBPA exposure.

### 3.2. Anti-RSV Activity of AF-08 or TBBPA *In Vitro*

AF-08 and TBBPA were examined for their direct anti-RSV activity by plaque reduction assays using HEp-2 cells (Table 1). In the assays, effective concentrations of ribavirin as a positive control were similar to the results reported by Konno et al. [31]. TBBPA did not significantly inhibit the plaque formation of RSV at 0.5 *μ*g/mL (1 *μ*M) to 16.3 *μ*g/mL (30 *μ*M), and no plaques were detected at 54.4 *μ*g/mL (100 *μ*M) because of the absence of more than 50% of uninfected cells. In the MTT assay, the percentage of viable cells in the presence of TBBPA at 54.4 *μ*g/mL was 54.9 ± 7.5%. In the case of AF-08, a slight plaque reduction was observed at 1 *μ*g/mL. However, plaques were not detected at 5 *μ*g/mL, because of the absence of more than 50% of uninfected cells. The MTT assay of AF-08 showed that the viable cells at AF-08 of 5 *μ*g/mL were 66.9 ± 7.2%. Thus the plaque reduction might be due to cytotoxicities of TBBPA and AF-08 rather than anti-RSV activity. TBBPA and AF-08 evidently did not show anti-RSV activity at their noncytotoxic concentrations.

### 3.3. Effects of AF-08 on Cytokine Production in BALF of RSV-Infected Mice Exposed to TBBPA

 It is known that the level of IFN-*γ*, a Th1 cytokine, in BALF of RSV-infected mice is a sensitive marker of the severity of RSV infection of lungs [11–13, 25]. Thus, we compared the IFN-*γ* levels in the BALF from RSV-infected mice exposed to TBBPA with and without AF-08 treatment on days 1 and 5 after infection. As shown in Table 2, on day 5 after infection, TBBPA exposure significantly increased IFN-*γ* level in the BALF of RSV-infected mice as compared with the control (*P* < 0.05 versus control by Student's *t*-test), but its combination with AF-08 was significantly effective in blocking the increase prophylactically (*P* < 0.05 versus TBBPA by Student's *t*-test). The blocked IFN-*γ* level was similar to the level in the BALF of RSV-infected mice treated with AF-08 alone. The IFN-*γ* levels in the AF-08-treated mice in spite of TBBPA exposure were significantly lower than that in the control (*P* < 0.05 versus control by Student's *t*-test). On day 1 after infection, IFN-*γ* levels with AF-08 alone and in combination with TBBPA tended to block the increase in the level with TBBPA alone, although the difference was not statistically significant. AF-08 was effective in prophylactically blocking the increase in IFN-*γ* level by TBBPA exposure in the lungs of RSV-infected mice.

To evaluate the effects of AF-08 on the immune system of RSV-infected mice with TBBPA exposure, the levels of pro-inflammatory cytokines (TNF-*α* and IL-6) and Th2 cytokines (IL-4 and IL-10) in BALF were also measured on days 1 and 5 after infection (Table 2). On day 1 after infection, TBBPA exposure significantly increased the TNF-*α* level in the BALF of RSV-infected mice (*P* < 0.05 versus control by Student's *t*-test). The combination with AF-08 significantly blocked the increase (*P* < 0.01 versus TBBPA by Student's *t*-test), and the prophylactically blocked level was similar to that in RSV-infected mice treated with AF-08 alone, although the former level in the combination was significantly lower than that in the control (*P* < 0.01 versus control by Student's *t*-test), while the latter level in AF-08 alone was not. In the case of IL-6, AF-08 also seemed to be effective in reducing the IL-6 levels in RSV-infected mice despite the TBBPA exposure on day 1. On day 5, the levels of TNF-*α* were much lower than those on day 1 and the effects of AF-08 on TNF-*α* level were not clear. However, AF-08 significantly reduced IL-6 levels in RSV-infected mice despite TBBPA exposure (*P* < 0.01 versus control or TBBPA by Student's *t*-test). AF-08 was effective in blocking TBBPA-dependent exacerbation in proinflammatory cytokine response. All RSV infections investigated in these studies caused a rise in levels of proinflammatory cytokines. In the cases of Th2 cytokines, IL-4 levels were low on days 1 and 5 after infection and the effects of AF-08 and/or TBBPA were not obvious. The IL-10 level in the RSV-infected control mice was not significantly affected by TBBPA exposure and/or AF-08 treatment, excepting the significant reduction of IL-10 level in AF-08-treated mice (*P* < 0.05 versus control by Student's *t*-test). Thus, AF-08 was mainly effective in controlling the rise of IFN-*γ*, TNF-*α*, and IL-6 levels caused by TBBPA exposure in RSV-infected mice.

### 3.4. Effect of AF-08 on Production of Anti-RSV Antibody in Mice Exposed to TBBPA

**Figure 2:**
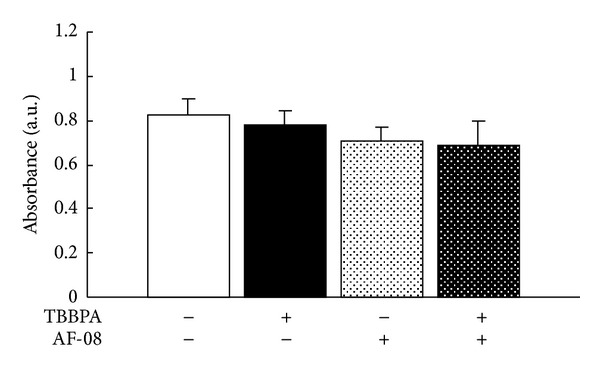
Effects of AF-08 on anti-RSV antibody production in RSV-infected mice with TBBPA exposure. Five mice in each group were fed a powdered diet mixed with 1% TBBPA alone, 0.02% AF-08 alone, or 1% TBBPA plus 0.02% AF-08 or without 1% TBBPA and 0.02% AF-08 (control) for 4 weeks and then infected with RSV at 10^6^ PFU/mouse. On day 5 after infection, the amounts of anti-RSV antibody in sera of RSV-infected mice were determined by ELISA. Bars indicate standard errors.

The effect of AF-08 on production of antibodies to RSV was compared in sera prepared from RSV-infected mice with and without TBBPA exposure. As shown in Figure 2, TBBPA exposure alone, AF-08 treatment alone, and their combination did not affect the anti-RSV antibody levels in sera of RSV-infected mice on day 5 after infection. Neither AF-08 treatment nor TBBPA exposure affected the production of anti-RSV antibody in RSV-infected mice.

### 3.5. Effect of AF-08 on Subpopulations of Immune Cells in BAL from RSV-Infected Mice

 To evaluate the effect of AF-08 on pulmonary immune responses in RSV-infected mice with TBBPA exposure, the subpopulations of immune cells in BALF were analyzed by flow cytometry on days 1 and 5 after infection (Table 3). Total numbers of BAL cells collected from an RSV-infected mouse in each group were 2.2 to 2.8 × 10^5^ cells, and there were no significant differences in the numbers of BAL cells in control, AF-08-treated, TBBPA-treated, and AF-08 plus TBBPA-treated groups (data not shown). On day 1, CD11b^+^ cells including macrophages and dendritic cells were dominant in BAL cells in each group (87.8%, 81.2%, 87.1%, and 87.3%), but the percentages of CD11b^+^ cells evidently decreased on day 5 (28.4%, 13.5%, 22.2%, and 17.6%). On the other hand, the percentages of CD8a^+^ cells (4.0%, 4.1%, 5.5%, and 4.5%) in each group on day 1 markedly increased on day 5 (50.8%, 62.5%, 69.1%, and 49.1%). However, TBBPA exposure and/or AF-08 treatment did not markedly affect the percentages of CD11b^+^ and CD8a^+^ cells in the controls on days 1 and 5. The ratios of percentages of CD11b^+^ cells in the groups of AF-08, TBBPA, and AF-08 and TBBPA against those in the control were 0.92 to 0.99 on day 1 and 0.62 to 0.83 on day 5. The ratios of percentages of CD8a^+^ cells in the groups of AF-08, TBBPA, and AF08 and TBBPA against those in the control were 1.03 to 1.38 on day 1 and 0.97 to 1.36 on day 5. AF-08 seemed to be effective in reducing the ratio of pulmonary CD8a^+^ cells in RSV-infected mice with TBBPA exposure.

## 4. Discussion

Propolis is a natural product known worldwide as a folk medicine and used as a dietary supplement because of its wide range of reported biological activities [1–6]. The chemical composition of propolis depends on the area and vegetation where it was harvested [6]. It has been administered orally for improvement of the symptoms of viral infection [5, 6]. However, there has been little research to elucidate the theoretical bases for the clinical efficacy of propolis. In this study, we characterized the basis of the biological efficacy of propolis on the exacerbation of pneumonia caused by TBBPA exposure in RSV-infected mice and found that a Brazilian propolis, AF-08, is effective in alleviating pneumonia exacerbated by TBBPA exposure in RSV-infected mice through limitation of excess cellular immune responses. Previously we showed that the oral administration of AF-08 is effective in ameliorating skin herpes symptoms in mice through activation of a delayed type hypersensitive reaction based on Th1 immunity as a major immune defense system in intradermal HSV-1 infection [6, 32]. In this study, AF-08 did not exhibit anti-RSV activity in a plaque reduction assay (Table 1) or affect virus titer in the lungs of RSV-infected mice (Figure 1). Thus, AF-08 may be characterized as possessing immunomodulatory activity based on cellular immunity. It may be worthwhile to further investigate the immunologically active compounds that are involved in AF-08.

TBBPA exposure significantly increased virus titers in the lungs of RSV-infected mice, while coadministration of AF-08 with TBBPA blocked this increase; virus titer in these mice was not different from that observed for controls (Figure 1). However, neither TBBPA nor AF-08 exhibited direct anti-RSV activity *in vitro* (Table 1). Thus, the activated immunological responses to eliminate infectious RSV particles and RSV-infected cells in the lungs of mice are probably responsible for the alleviation of pneumonia exacerbated by TBBPA exposure. Perinatal exposure to BFRs was also shown to exacerbate pneumonia in RSV-infected offspring mice with an increase in pulmonary viral titers and there was a correlation between the severity of RSV infection in the offspring mice and the dose exposed to pregnant mice [12, 13]. The dose (30 mg/kg/day) of AF-08 given to mice was analogous to that ingested by humans [6]. From the viewpoint of environmental medicines, it is possible that oral administration of AF-08 not only to adults but also to children born from women exposed to BFRs such as TBBPA is prophylactically useful to prevent the exacerbation of RSV infection.

IFN-*γ*  has been reported to be a potent stimulator of lymphocyte migration into tissues and a major mediator of lymphocyte recruitment in Th1 immunity [33]. The level of IFN-*γ*, a marker of pneumonia, has been shown to reach a maximum on day 5 after infection in RSV-infected mice, and its production was enhanced in TBBPA-exposed mice [25]. In this study, the elevation of the IFN-*γ* level by TBBPA exposure in the lungs of RSV-infected mice on day 5 was confirmed. The elevation by TBBPA on day 5 was significantly reduced by AF-08 in combination with TBBPA (Table 2). Also, levels of both proinflammatory cytokines (TNF-*α* and IL-6) in RSV-infected mice with TBBPA exposure on day 1 were reduced by AF-08 in the combination with TBBPA (Table 2). The reductions in TNF-*α* and IL-6 by AF-08 were prominent early in the infection, suggesting that the beneficial effect of AF-08 was due to the suppression of inflammation early after infection. In addition, AF-08 was effective in markedly reducing the IFN-*γ* level on day 5 (Table 2). Thus, the suppression of proinflammatory cytokine production by AF-08 early in the infection was associated with weakening inflammation in the lungs of infected mice and reducing the infiltrated cell population quantitatively and/or qualitatively, resulting in suppression of the overreaction of Th1 immune response correlated with the elevation of IFN-*γ* level in the infected lungs. Actually, AF-08 alone was effective in reducing the levels of proinflammatory cytokines (TNF-*α* and IL-6) in RSV-infected mice on day 1 and the level of IFN-*γ* on day 5. Therefore, the suppression of proinflammatory cytokine productions by AF-08 in RSV-infected mice is probably responsible for the reduction of the IFN-*γ* level in the combination leading the alleviation of pneumonia. Also, as the excess productions of proinflammatory cytokines by TBBPA in RSV-infected mice caused severe inflammatory response, the suppression of the cytokines by AF-08 was suggested to be effective in blocking an imbalance in the inflammatory response caused by TBBPA in RSV-infected mice. TBBPA was reported to activate the MAP kinases and protein kinase C [34, 35]. AF-08 may be effective in suppressing their activation in signal transduction systems involved in the overexpression of proinflammatory cytokines.

TBBPA exposure failed to significantly elevate the levels of Th2 cytokines (IL-4 and IL-10) in RSV-infected mice on either day 1 or day 5, and there were no significant reductions of the Th2 cytokine levels by AF-08 in combination with TBBPA (Table 2). TBBPA and AF-08 did not noticeably affect Th2 cytokine (IL-4 and IL-10) levels in RSV-infected mice. It was reported that an imbalance of Th1/2 cytokines in the immune system is involved in the severity of RSV-induced disease [36]. The strong production of Th1 cytokines, such as IFN-*γ*, exacerbating RSV infection might disorder Th2 cytokine production in infected mice after TBBPA exposure and/or AF-08 treatment. In RSV-infected mice, TBBPA and/or AF-08 had no effect on the production of anti-RSV antibody (Figure 2), suggesting that neither TBBPA nor AF-08 mainly affects humoral immunity. However, TBBPA and AF-08 affected cellular immunity induced by Th1 immune response associated with IFN-*γ* production in RSV-infected mice, as described before. It is possible that TBBPA produced an imbalance between the Th1 and Th2 immunity caused by excessive Th1-dominant immune response in RSV-infected mice and AF-08 blocked the imbalance by limiting the excessive Th1 immunity.

FACS analysis showed that CD11b^+^ cells involving macrophages and dendritic cells and CD8^+^ cells involving cytotoxic T cells were dominant in BAL cells from RSV-infected mice in spite of TBBPA exposure and/or AF-08 treatment on days 1 and 5, respectively (Table 3). CD11b^+^ cells and CD8^+^ cells are the major producers of proinflammatory cytokines and IFN-*γ*, respectively. Thus, the results of FACS analysis were consistent in the elevations of proinflammatory cytokine production on day 1 and IFN-*γ* production on day 5 (Table 2). TBBPA exposure and/or AF-08 treatment had no remarkable effect on the percentages of CD11b^+^ cells and CD8^+^ cells in BAL cells from RSV-infected mice (Table 3). However, while TBBPA was effective in elevating the productions of proinflammatory cytokines and IFN-*γ*, AF-08 was effective in reducing their productions (Table 2). TBBPA has been suggested to induce a qualitative change in immune cells that work in the early phase of RSV infection [25]. Thus, AF-08 also might induce a qualitative change in immune cells in the lungs of RSV-infected mice.

We characterized the experimental bases of Brazilian propolis AF-08 treatment using an RSV infection model in mice. Together, all results indicate that propolis AF-08 showed immunological activity against intranasal RSV infection in mice. In particular, the immunological activity associated with the limitation of IFN-*γ* production inducing Th1 immunity in mice may contribute to the elucidation of various pharmacological actions of propolis AF-08 in health and disease.

## Figures and Tables

**Table 1:** Anti-RSV activity and cytotoxicity of TBBPA and AF-08 *in vitro*.

	*μ*g/mL	% of viable cells in MTT assay	% of plaque numbers in plaque reduction assay
TBBPA	0	100	100
	0.5	102.0 ± 7.9	95.2 ± 24.4
	1.6	94.8 ± 3.5	87.1 ± 8.4
	5.4	98.8 ± 5.6	91.9 ± 9.7
	16.3	86.9 ± 1.3	80.6 ± 10.1
	54.4	54.9 ± 7.5	++*

AF-08	0	100	100
	0.1	—	93.8 ± 8.1
	0.5	—	82.8 ± 31.2
	1	—	74.2 ± 3.6
	5	66.9 ± 7.2	++*
	20	56.5 ± 5.3	—
	40	33.0 ± 1.7	—

Ribavirin	0	—	100
	1	—	75.0 ± 12.5
	5	—	29.2 ± 20.1
	10	—	10.4 ± 7.5
	15	—	0

Values are mean ± standard deviation (SD) of the mean of three to five independent experiments.

(—) indicates that experiments were not done.

*% of plaque numbers could not be determined, because of the absence of more than 50% of uninfected and stained cells from the surface of dishes.

**Table 2:** Effect of AF-08 on cytokine concentrations in BALF from RSV-infected mice exposed or unexposed to TBBPA.

Cytokines	Concentrations ± SE (pg/mL) in BALF			
	Control	AF-08	TBBPA	TBBPA + AF-08
Day 1				
IFN-*γ*	23.9 ± 4.3	30.0 ± 5.3	56.2 ± 10.9	33.1 ± 6.8
TNF-*α*	271.6 ± 25.2	148.1 ± 76.2^c^	339.0 ± 22.3^a^	122.1 ± 17.9^b,d^
IL-6	1076.1 ± 150.9	848.7 ± 341.7	926.1 ± 183.8	<470.0 ± 305.3
IL-4	<28.0 ± 11.4	<20.0 ± 0.0	<28.0 ± 11.4	<20.0 ± 0.0
IL-10	144.0 ± 63.1	149.4 ± 45.4	208.7 ± 69.9	102.3 ± 35.6
Day 5				
IFN-*γ*	2557.4 ± 117.4	1507.6 ± 491.9^a,c^	3348.3 ± 410.8^a^	1108.3 ± 511.3^a,c^
TNF-*α*	<40.0	<40.0	<40.0	<40.0
IL-6	111.8 ± 26.7	<28.7 ± 15.0^b^	<67.7 ± 60.9	<20.0 ± 0.0^b^
IL-4	<20.0 ± 0.0	<20.0 ± 0.0	<20.6 ± 1.0	<36.4 ± 28.3
IL-10	197.4 ± 37.4	118.2 ± 9.5^a^	195.5 ± 99.2	110.7 ± 68.1

BALF was collected from 3-4 mice in each group on day 1 and day 5 after infection. Each BALF was analyzed to determine cytokine concentrations by ELISA. ^a^ *P* < 0.05 versus control by Student's *t*-test. ^b^ *P* < 0.01 versus control by Student's *t*-test. ^c^ *P* < 0.05 versus TBBPA by Student's *t*-test. ^d^ *P* < 0.01 versus TBBPA by Student's *t*-test.

**Table 3:** Effect of AF-08 on subpopulations in BAL cells of RSV-infected mice exposed or unexposed to TBBPA.

Subpopulation	% of total cell populations			
	Control	AF-08	TBBPA	TBBPA + AF-08
Day 1				
CD11b^+^	87.8	81.2	87.1	87.3
CD8a^+^	4.0	4.1	5.5	4.5
Day 5				
CD11b^+^	28.4	23.5	22.2	17.6
CD8a^+^	50.8	52.5	69.1	49.1

BAL cells were collected from 3-4 mice in each group on day 1 and day 5 after infection and pooled by group. The subpopulations of pooled BAL cells were analyzed by FACS.
